# Improving the performance of β-turn prediction using predicted shape strings and a two-layer support vector machine model

**DOI:** 10.1186/1471-2105-12-283

**Published:** 2011-07-13

**Authors:** Zehui Tang, Tonghua Li, Rida Liu, Wenwei Xiong, Jiangming Sun, Yaojuan Zhu, Guanyan Chen

**Affiliations:** 1 Department of Chemistry, Tongji University, Shanghai, 200092, China

## Abstract

### Background

The β-turn is a secondary protein structure type that plays an important role in protein configuration and function. Development of accurate prediction methods to identify β-turns in protein sequences is valuable. Several methods for β-turn prediction have been developed; however, the prediction quality is still a challenge and there is substantial room for improvement. Innovations of the proposed method focus on discovering effective features, and constructing a new architectural model.

### Results

We utilized predicted secondary structures, predicted shape strings and the position-specific scoring matrix (PSSM) as input features, and proposed a novel two-layer model to enhance the prediction. We achieved the highest values according to four evaluation measures, i.e. Q*_total _*= 87.2%, MCC = 0.66, Q*_observed _*= 75.9%, and Q*_predicted _*= 73.8% on the BT426 dataset. The results show that our proposed two-layer model discriminates better between β-turns and non-β-turns than the single model due to obtaining higher Q*_predicted_*. Moreover, the predicted shape strings based on the structural alignment approach greatly improve the performance, and the same improvements were observed on BT547 and BT823 datasets as well.

### Conclusion

In this article, we present a comprehensive method for the prediction of β-turns. Experiments show that the proposed method constitutes a great improvement over the competing prediction methods.

## Background

The β-turn is a secondary protein structure type that plays an important role in protein configuration and function. A β-turn consists of four consecutive residues in a protein chain that does not form an α-helix, and the distance between *C*_α_(*i*) and *C*_α_(*i*+3) is less than 7 Å, where *C*_α_(*i*) denotes the alpha-carbon of an amino acid residue [1,2]. On average, β-turns account for approximately 25% of the globular protein residues [3]. β-turns are usually described as the orienting structure, because they orient α-helices and β-sheets, defining indirectly the topology of proteins. They are also involved in the biological activity of peptides as the bioactive structures that interact with other molecules, such as receptors, enzymes, or antibodies [4]. Formation of the β-turn is also a vital stage during the process of protein folding [5]. Therefore, development of accurate prediction methods to identify β-turns in protein sequences would provide valuable insights and inputs for fold recognition and drug design, and it would meet the heavy demand by reducing the experiment time and cost.

The β-turn prediction methods developed from the beginning until now can be divided into two categories: those based on statistical methods and those based on machine-learning approaches. In statistical approaches, a series of positional frequencies and conformational parameters are derived from each position in β-turns. They include the Chou-Fasman method [6], Thornton's algorithm [7], GORBYURN [8], the 1-4 & 2-3 correlation models [9], and the sequence-coupled model [10]. More recently, the COUDES method based on propensities and multiple alignments has been proposed [11]. The position-specific scoring matrix (PSSM), which is calculated with PSI-BLAST [12] and secondary structure information predicted by PSIPRED [13] and SSPRO2 [14] were utilized by COUDES to improve the accuracy of prediction. The second category, based on machine-learning approaches, includes BTPRED [15], BetaTPred2 [16,17], and MOLEBRNN [18], which are based on artificial neural networks (ANN), Kim's method [19] based on K-nearest neighbor (KNN), as well as the most recent prevailing method based on support vector machines (SVMs) [4,20-28]. Inclusion of secondary structure information and PSSM in ANNs and SVMs has been shown to improve prediction performance [29,30]. The best SVM-based predictor according to Q*_total _*that was developed by Zheng and Kurgan utilized window-based information extracted from four predicted, three-state secondary structures, together with a selected set of PSSM values as an input [27]. They achieved the following results: Q*_total _*= 80.9%, Q*_observed _*= 55.6% and MCC = 0.47. However, the quality of the prediction is still a challenge, and there is substantial room for improvement.

In this paper, we propose a comprehensive method for protein β-turn prediction. Our innovations focus on discovering effective features and constructing a new architectural model. Besides generating effective features, a two-layer SVM model based on a clustering approach is proposed in this paper. Seven-fold cross validation tests on the BT426 dataset achieve a result of Q*_total _*= 87.2%, Q*_observed _*= 75.9% and MCC = 0.66, which demonstrate that the proposed approach can achieve significant improvement over the competing β-turn prediction methods.

## Methods

### Datasets

The dataset of 426 protein sequences (denoted by BT426), which was developed by Guruprasad and Rajkumar [4], was chosen to train and test our method. The structure of each protein chain in this dataset has been determined by X-ray crystallography at better than 2.0 Å resolution, and no two protein chains have > 25% identity. The program PROMOTIF [31] was implemented to identify the observed β-turns in these crystal structures. Each chain contained at least one β-turn. After finding the optimal input sheme and kernel parameters, we utilized two additional datasets to validate the performance of the method. The datasets consist of 547 and 823 protein chains and are denoted as BT547 and BT823, respectively. They were constructed using PDBSELECT list published in June 2000 and October 2003 [32], respectively, by Fuchs and Alix [33]. They share the same characteristics as BT426 dataset.

### Features

#### PSSMs

In the proposed approach, PSSMs are utilized as input features, since they have been shown to contribute significantly to the accuracy of β-turn prediction [27-30]. The PSSM is computed using two rounds of PSI-BLAST searches against NCBI non-redundant (nr) amino acid sequence databases, with default parameters [12]. The PSSM is a matrix of N×20 elements, where N is the number of residues of the query sequence. The PSSM values are scaled within the range [0 1] using the standard logistic function:(1)

where x is the matrix value that stands for the propensity of that particular residue substitution at that position.

#### Predicted secondary structures

Three secondary structure predictors, PHD [34], Jpred [35], and PROTEUS [36], are considered in this paper. PHD and Jpred are based on the amino acid sequence, while PROTEUS is developed by using both sequence and structural alignment. As the size of the protein sequence database gets larger and larger, the probability of a newly identified sequence having a structural homologue is actually high. Experiments show that PROTEUS achieves higher prediction accuracy on the BT426 dataset than the other two predictors. Therefore, we utilized PROTEUS to predict secondary structure in our final model.

The protein secondary structures were predicted as three structures: helix, strand and coil. The predicted secondary structure information of each residue was encoded as: helix→ (1 0 0), strand→ (0 1 0), coil→ (0 0 1).

#### Predicted shape strings

**Figure 1:**
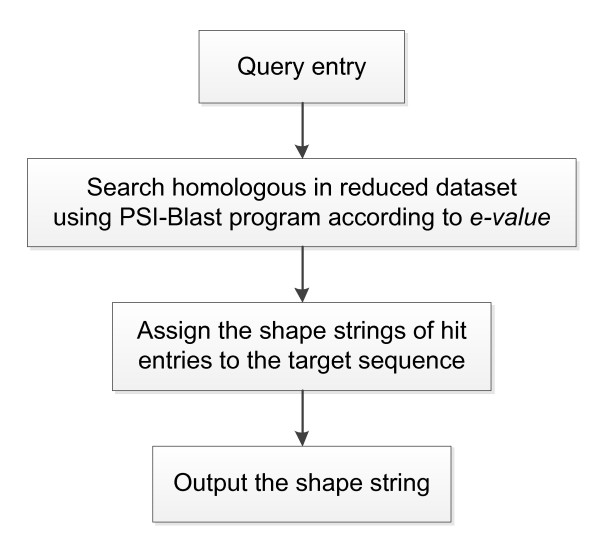
**The flow chart of shape string prediction**.

Since the classical three-state secondary structure did not indicate precisely the backbone protein structure, another type of one-dimensional string of symbols representing the clustered regions of Φ, Ψ torsion angle pairs, called shape string [37], was considered as a new feature of our predictor. Predicted dihedral angles have been applied successfully for secondary structure prediction [38,39], β-turn prediction [26] and three-dimensional structure of protein fragments [40]. Recently, shape string was successfully used in gamma-turn prediction [41]. In this work, shape strings were predicted from a predictor constructed based on structural alignment approach and shown useful in predicting β-turns. Shape strings were represented by eight states, i.e. S, R, U, V, K, A, T and G. For a sequence in the PDB database, the shape string can be calculated according to its three dimensional structures determined by experimental measurements. For a sequence whose structure is unknown, the shape string can be predicted using amino acid sequence information. Here we constructed a shape string predictor based on structural alignment (as shown in Figure 1).

We constructed a non-redundant dataset of nr-PDB (Sep. 2010) (download from ftp://ftp.ncbi.nlm.nih.gov/blast/db), cutting at 30% sequence identity containing 13609 protein entries using CD-HIT [42] (426 sequences were removed from the original dataset). The shape string of each entry was obtained from the web server [43].

For a given target sequence, the PSI-BLAST program [12] was carried out on the reduced dataset to find its homologous sequences. Then, matched sequences whose e-value was below a given threshold (1 × 10^-5^) were ranked according to the e-value in ascending order. Those sequences were judged one by one. The shape string of the matched part of the sequence was assigned to the target sequence. Only the unmatched part of the target sequence was considered when matching with the next ranked sequence. When finishing the assignment, there would still be some unmatched parts of the target sequence, and therefore, this part would have no shape string information. At the encoding stage, we used × to represent those empty positions. Nine characters of shape string information were encoded in an orthogonal manner, e.g. S→ (1 0 0 0 0 0 0 0 0), R→ (0 1 0 0 0 0 0 0 0).

### Two-layer scheme

**Figure 2:**
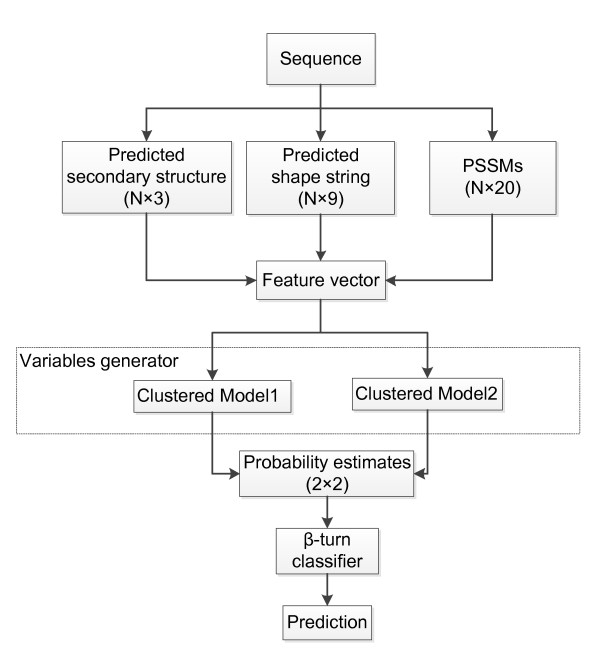
**The architecture of the proposed prediction method**. N denotes the window size.

The overall architecture of the proposed system is shown in Figure 2. We built a two-layer SVM predictor using the probability estimates of all samples from two clustered models in the first layer as the input of the second layer.

Features mentioned above were calculated when a window of 8 AA sliced from the N-terminal end to the C-terminal end of a protein sequence. Each window was tagged with a label of β-turn (positive) or non-β-turn (negative), according to whether consecutive, centered four residues form a β-turn or not. Feature vectors were fed into two clustered models to compute the probability estimates. Then, the probability estimates of each sample from two models were combined as the input of a second-layer SVM predictor to make a final prediction.

#### Clustered model

The remarkable feature of our approach is the clustered model. In our trial experiments, we found that if the positive set was divided into a few subsets by a suitable clustering algorithm, the prediction accuracy of N-fold cross validation of each positive subset with randomly selected negative samples was promoted significantly when the ratio of positive to negative was kept the same (i.e. 1:3). We conjecture that this is because the distribution of the positive samples in a subset is centralized and compacted, and it means that good performance would be expected when a multi-model could be used in the whole prediction. However, there was still a barrier to overcome at this stage. That is, one does not know which model should be used in practice when predicting unknown samples. If an incorrect subset model is used, the performance would be unsatisfactory. Therefore, we used these clustered models as variable generators, and, furthermore, constructed a two-layer learning machine.

**Figure 3:**
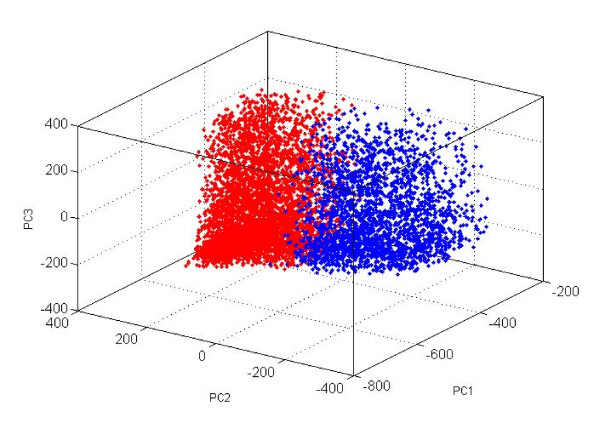
**The distribution of two clusters**. The axes correspond to top 3 PCs (×100) of PCA (principal component analysis) of positive samples with β-turns. Red dots denote samples in cluster 1. Blue dots denote samples in cluster 2.

At the very beginning, the whole positive set was divided into two subsets by a K-means clustering algorithm using original variables. The distribution of those two clusters is shown in Figure 3. The two centralized and compacted subsets are utilized to build SVM models with randomly selected negative samples, whose size is three times the positive subsets. The two clustered models, of course, cannot be utilized directly in the prediction, but they can be considered as variable generators, and are named "clustered model 1" and "clustered model 2." During training and prediction stages, these clustered models are unchanged, and all the samples enter both clustered models. Probability estimates of samples for being positive/negative are generated for the next step. Such variable generators often appear in published papers, for example the PHD predictor [34] for protein secondary structure, in which the first layer of ANN is just a variable generator, and the probabilities of three states are outputs for the next layer. We clearly understand that, for one clustered model the output probability estimates for being positive/negative are not all correct; however, there are always some correct pairs. The judgment and weighting task is left to next layer SVM modeling.

#### Secondary layer modeling

As mentioned above, two clustered models in the first layer were utilized as variable generators to generate probability estimates. Therefore, the input of the second-layer predictor was a 2 × 2 vector. Seven-fold cross validation was used to perform tests on the dataset. The folds were created by randomly selecting equal numbers of samples which were produced by the sliding window technique. Each sample was only predicted once, and never involved in corresponding model construction of both layers. The overall prediction accuracy is the percentage of correctly predicted samples.

In this work, we employed the support vector machine (SVM) [44] classifier in both layers. LibSVM [45], a popular SVM software package, was employed for the training and testing of the SVM classifiers. RBF SVM was used in our prediction. Two parameters, C and gamma, were optimized using the default grid-search approach to achieve the optimized predictive performance. The optimized parameters (C, gamma) for clustered model 1 and clustered model 2 were both (0.5, 0.0078125). That for secondary layer model was (32, 8).

### Evaluation

Four measures, Q*_total_*, Q*_predicted_*, Q*_observed_*, and MCC are commonly used to evaluate the quality of prediction [11]. During the cross validation test, the confusion matrix, which comprises true positive (TP), false positive (FP), true negative (TN) and false negative (FN) was used to calculate these measures:

1) Q*_total _*is defined as the percentage of correctly classified β-turns(2)

2) Q*_predicted _*is the percentage of correctly predicted β-turns among the predicted β-turns(3)

3) Q*_observed _*is the percentage of correctly predicted β-turns among the observed β-turns(4)

4) Matthew's Correlation Coefficient (MCC) [46] is calculated(5)

The MCC value takes account of both over- and under-predictions and is between -1 and 1.

We also adopt the receiver operating characteristics (ROC) and report the area under the ROC curve to measure the ability of a method to correctly classify β-turns and non-β-turns.

## Results and Discussion

### Comparison with competing prediction methods

The selected features, i.e. PSSMs, secondary structures predicted by PROTEUS and shape string predicted by our structural alignment approach, and two-layer SVM scheme were applied in the proposed prediction model. The 7-fold cross validation test results on the BT426 dataset are summarized and compared with competing methods in Table 1. The results are organized in descending order by the values of Q*_total_*.

**Table 1:** Comparison of the proposed and the competing methods on the BT426 dataset

Predictor	Q*_total _*	MCC	Q*_observed _*	Q*_predicted _*
**This paper**	**87.2**	**0.66**	**75.9**	**73.8**
Zheng and Kurgan [27]	80.9	0.47	55.6	62.7
Hu and Li [24]	79.8	0.47	68.9	55.6
DEBT [26]	79.2	0.48	70.1	54.8
BTSVM [21]	78.7	0.45	62.0	56.0
MOLEBRNN [18]	77.9	0.45	66.0	53.9
Zhang et al. [20]	77.3	0.45	67.0	53.1
BETAPRED2 [17]	75.5	0.43	72.3	49.8
Kim [19]	75.0	0.40	66.7	46.5
COUDES [11]	74.8	0.42	69.9	46.5
BTPRED [30]	74.4	0.35	57.3	48.3

Note: Results of other β-turn prediction methods are obtained from the paper which proposed DEBT method.

Table 1 shows that the proposed method achieved the highest values according to four evaluation measures on the BT426 dataset. The Q*_total _*was 6.3% higher than that obtained with Zheng and Kurgan's method, which was based on an ensemble of predicted secondary structures and multiple alignments, and was the first to break the 80% barrier among the existing competing methods. The MCC value of the proposed method was 0.19 higher than their method. The Q*_observed _*and Q*_predicted _*were higher by 20.3% and 11.1%, respectively. In comparison with the most recently developed method (i.e. the DEBT method that predicts β-turns from multiple sequence alignments, predicted secondary structures, and for the first time, predicted dihedral angles), the Q*_total _*and MCC of our method were higher by 8.0% and 0.18, respectively. This outstanding result indicates that the proposed prediction model can better discriminate between β-turns and non-β-turns when compared with the competing methods.

### The effect of two-layer scheme

The proposed two-layer scheme is different from existing two-stage classifiers for protein structure prediction [47-49]. We built the two-layer model based on a clustering approach. The probability estimates of first-layer models are fed to the second layer to make a final prediction. We performed several experiments using different feature combinations, as well as a single SVM predictor. The results are shown in Table 2. The Q*_total _*of the two-layer model is 2.8% higher than the single model when using three predicted secondary structures and PSSMs as input features. The Q*_predicted _*is higher by 7.2%. When using predicted secondary structures from PROTEUS instead, the Q*_total _*of the two-layer model was 1.9% higher than the single model, while the Q*_predicted _*was higher by 6.2%. In comparison with predictors using the same kind of information, the Q*_total _*of the two-layer model was higher than the best value as reported [27]. Two-layer model can also achieve higher value of Q*_predicted_*. Higher Q*_predicted _*value means that a larger fraction of the predicted β-turns are in fact β-turns. This indicates that the two-layer model can better discriminate between β-turns and non-β-turns than the single model. We note that the MCC value was lower when the two-layer model was applied but still higher than the best value as reported [26]. To obtain higher predictive accuracy and lower false positive rate, we preferred the two-layer model in this paper. When predicted shape string was incorporated into the input features, both single model and two-layer model achieved great improvements. In this situation the Q*_total _*of the single model reached 87.3%, 0.1% higher than that of the two-layer model. The Q*_predicted _*of two-layer model was 4.0% higher than the single model. The comparison between single and two-layer model on MCC value remained the same as we mentioned above.

**Table 2:** Results of different sets of features performed on both single and two-layer models

Features	Predictor	Q*_total _*	MCC	Q*_observed_*	Q*_predicted _*
PSS (PHD, Jpred, PROTEUS) PSSMs	Single	78.3	0.52	79.7	54.5
	Two-layer	81.1	0.51	64.8	61.7

PSS (PROTEUS) PSSMs	Single	80.8	0.58	84.1	58.0
	Two-layer	82.7	0.55	69.5	64.2

PSS (PROTEUS) PSSMs Predicted Shape Strings	Single	87.3	0.69	86.5	69.8
	Two-layer	87.2	0.66	75.9	73.8

Note: PSS refers to predicted secondary structure.

### Performance on additional datasets

Besides BT426 dataset used for training and testing our method, we utilized two additional datasets, i.e. BT547 and BT823 datasets, to validate the performance of the method. The obtained results show that when using the predicted secondary structures from PROTEUS, predicted shape strings and PSSMs as input features, both single model and two-layer model achieved high overall accuracy. Since the single model was less time-consuming, we chose the single model with those input features to perform tests on additional datasets. Shape strings were predicted by our structural alignment approach against non-redundant dataset of nr-PDB cutting at 30% sequence identity after removing identical sequences of BT547 and BT823 dataset. The parameters C and Gamma were set to the same as that optimized from BT426 dataset. Seven-fold cross validation was performed. The predictive performance of our method with other competing methods on BT547 and BT823 dataset was compared in Table 3. Our achieved results were the best around the methods reported to date. All four measures were remarkably higher than other methods.

**Table 3:** Comparison with other competing methods on additional datasets

Dataset	Predictor	Q*_total_*	MCC	Q*_observed_*	Q*_predicted_*
BT547	this paper	87.3	0.69	86.5	69.8
	DEBT [26]	80.0	0.49	68.7	55.9
	Zheng and Kurgan [27]	80.5	0.45	54.2	61.6
	COUDES [11]	74.6	0.42	70.4	48.7
	Hu and Li [24]	76.6	0.43	70.2	47.6
BT823	this paper	88.7	0.73	88.1	72.6
	DEBT [26]	80.9	0.48	66.1	55.9
	Zheng and Kurgan [27]	80.6	0.45	54.6	60.8
	COUDES [11]	74.2	0.41	69.6	47.5
	Hu and Li [24]	76.8	0.45	72.3	53.0

Note: Results of other β-turn prediction methods are obtained from the paper which proposed DEBT method.

### More accurate predicted secondary structures

Throughout the preceding research on β-turn prediction, predictors based on machine learning method emphasize selecting proper features to improve prediction performance. Now secondary structures and PSSMs are widely used in the predictions, and have been proven to be the most helpful features. It is possible to improve the accuracy of β-turn prediction using more accurately predicted secondary structures, for example, in this work, PROTEUS [36].

We observed that when using predicted secondary structures from PROTEUS, four evaluation measures of both the single model and the two-layer model were better than using three integrated predicted secondary structures. We calculated the accuracy of secondary structure prediction on the BT426 dataset performed by three predictors, i.e. PHD, Jpred and PROTEUS. The accuracy of PROTEUS on the BT426 dataset was 82.0%, which was 2.4% higher than Jpred, and 3.1% higher than PHD. PROTEUS performs structure-based sequence alignments as part of the secondary structure prediction process. It attained high prediction accuracy by integrating structural alignment with conventional (sequence-based) secondary structure methods. This observation indicates that by mapping the structure of a known homologue onto the query protein sequence, it is possible to predict a portion of the structure of the query protein's structure.

### Newly introduced predicted shape strings

**Figure 4:**
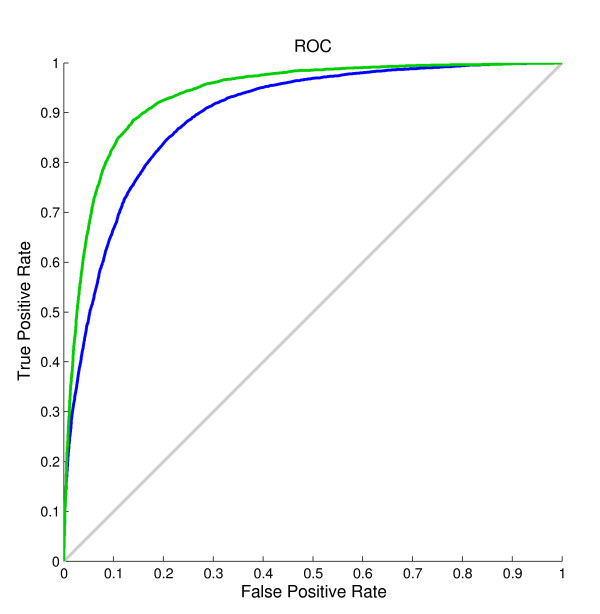
**ROC curves for the prediction on the BT426 dataset**. Green curve corresponds to the prediction using predicted secondary structures from PROTEUS, PSSMs and predicted shape strings as input features, while the blue curve corresponds to the prediction using predicted secondary structures from PROTEUS and PSSMs.

The results in Table 2 show that when the shape string predicted by our method was introduced into the model, the accuracy of both the single model and the two-layer model improved significantly. When using the predicted shape string as the only input feature, the single model achieved a result of Q*_total _*= 85.3%, MCC = 0.65, Q*_observed _*= 83.5%, and Q*_predicted _*= 66.3%. These results already outperformed other existing predictors. This was mainly because shape strings contain much richer conformation than secondary structures, and the precise protein structure could be reconstructed from shape strings [50]. Figure 4 illustrates the ROC curves for β-turn prediction before and after using predicted shape strings on the BT426 dataset. The improvement of corresponding areas under the curves (AUC) highlighted the effect of predicted shape strings. The AUC value after using predicted shape string is 0.94, 0.05 higher than that before using predicted shape strings.

**Figure 5:**
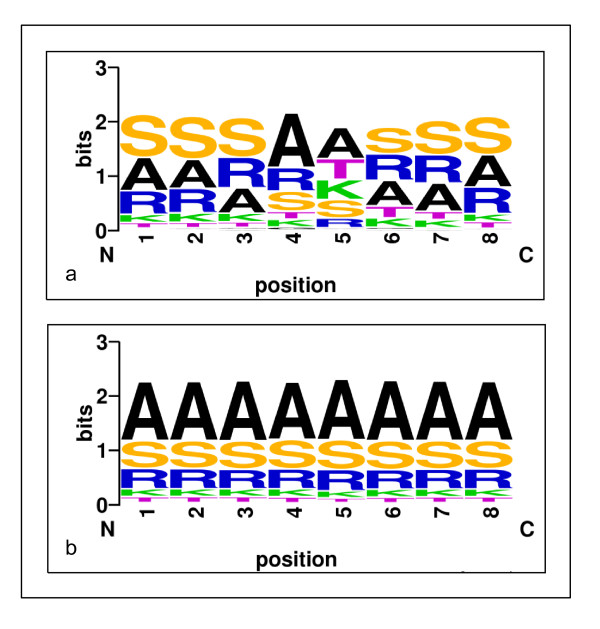
**The distribution of shape strings in sliding-window fragments of β-turns and non-β-turns**. (a) denotes the distribution in β-turns, while (b) denotes that in non-β-turns. The height of symbols indicates the relative frequency of that type of shape string at that position. Both were created by WebLogo [53].

**Figure 6:**
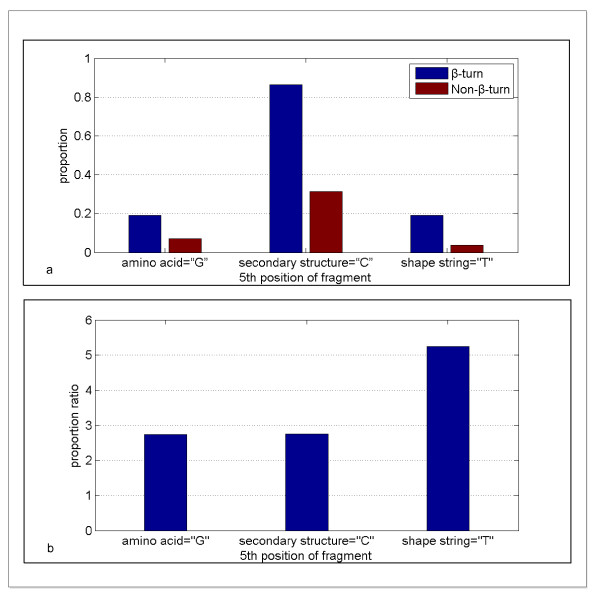
**The proportion ratio of G (Glycine), C (coil) and T (turns) existing in β-turns and non-β-turns**. (a) denotes the proportion of each type existing in β-turns and non-β-turns. (b) denotes the proportion ratio of β-turns to non-β-turns.

In our approach the predicted shape strings were of great role undoubtedly, we inferred that there was a strong relationship between shape strings and β-turns. In Figure 5 the distributions of shape strings of sliding window fragments are shown for both β-turns and non-β-turns. This indicates that the distributions of shape strings in β-turns and non-β-turns are quite different. There are three types of shape strings, A (α-helices), S (β-sheets) and R (poly Pro II), which occupy a great proportion in both β-turns and non-β-turns. However, T (turns, also called right-handed helix,) and K (3_10-helices) represents a large percentage in the 5th and 6th positions of β-turn fragments. We analyzed the 5th position of fragments. The proportion of G (Glycine, amino acid), C (coil, secondary structure) and T (turns, shape string) which are relatively rich in their types existing in the 5th position of β-turns and non-β-turns are shown in Figure 6(a). Figure 6(b) denotes the proportion ratios of β-turns to non-β-turns. It is obvious that the proportion ratio of T between β-turns and non-β-turns is larger than that of C and G. This great difference indicates the reason that the shape string feature performed much better than those predictors without shape string.

**Figure 7:**
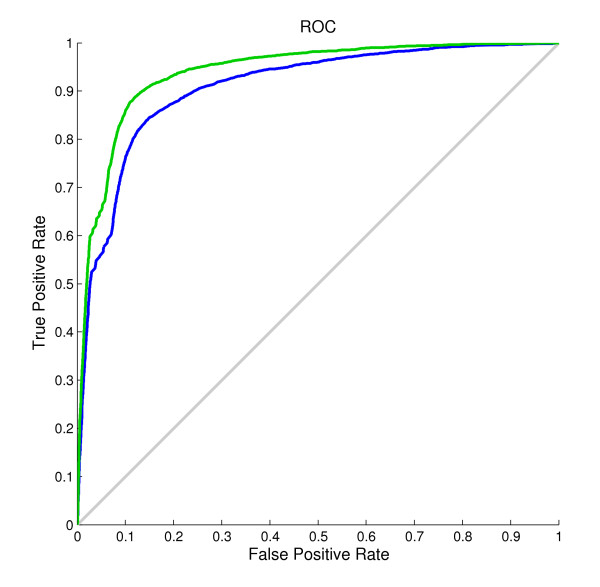
**ROC curves for the prediction using predicted shape string and real shape string**. Green curve corresponds to the prediction using real shape string, while the blue curve corresponds to the prediction using predicted shape string.

The DEBT method predicted β-turns using predicted backbone dihedral angles and secondary structures. The dihedral angles employed in this method were predicted by DISSPred [51] using a partition of seven clusters, which is similar to shape strings. DISSPred utilizes PSSMs as its input features, while our method predicted shape strings using structural alignment approach with the help of PSI-BLAST program. Through our simple shape string assignment approach, the accuracy of the shape string prediction was 79.4%. There is still a room for improving the shape string prediction. We did experiments by using real shape strings as the input feature. It achieved a result of Q*_total _*= 88.0%, MCC = 0.71, Q*_observed _*= 88.3% and Q*_predicted _*= 70.8%. The ROC curves for β-turn prediction using predicted shape strings and real shape strings were illustrated in Figure 7. The corresponding areas under the curves were 0.91 and 0.94, respectively. The gap of the results between predicted shape strings and real shape strings indicates that more accurate β-turn prediction will be achieved when we improve the accuracy of the shape string prediction.

A recent survey has found that less than 3% of new protein structures deposited into the PDB have a totally novel fold [52]. In other words, the vast majority of newly solved proteins could find homologues from pre-existing sequences in the PDB. In fact, for a given target sequence, there is a slight probability that we could not find any homologues from our reduced dataset. This will be our focus in the near future. In situations where no homologue is found, or only a portion of the query sequence could be predicted by the structural alignment method, a sequence-based shape string predictor could be constructed to cover the unpredicted portion. In other words, we could generate profiles using a window-based segment-matching approach. Machine learning methods will be utilized to make a prediction. We believe that these methods will better predict shape strings and further enhance protein structure prediction.

## Conclusions

In this article, we presented a comprehensive method for the prediction of β-turns. Our method utilized predicted secondary structures, predicted shape strings and PSSMs as input features, and proposed a novel two-layer model to enhance the prediction. The Q*_total _*of 87.2%, achieved for β-turn/non-β-turn prediction on the BT426 dataset, is 6.3% higher than the second best method. All other measures, the MCC of 0.66, the Q*_observed _*of 75.9% and the Q*_predicted _*of 73.8%, are also significantly higher than other methods. These results show that our method is more accurate than other β-turn prediction methods. It has been proven that the introduction of predicted shape string contributed significantly to our improvements. Moreover, the new architectural two-layer model is quite useful when a single model cannot achieve remarkable performance and can better discriminate between β-turns and non-β-turns due to obtaining higher Q*_predicted _*which means lower false positive rate. To further improve the predictions, we will focus in the future on obtaining a more precise shape string prediction.

Overall, protein structure prediction has come into a new stage based on structural alignment strategy. Several predictors based on structural alignment were developed for prediction of protein structure recently, for example, PROTEUS [36] for secondary structure prediction, and Frag1D [50] for one-dimensional protein structure prediction. In this study, shape string predictor was also based on structural alignment approach. With the growing protein structure databases, we believe that structural alignment approach will be the mainstream and make great progress in protein structure prediction in the future.

## Availability

The shape string predictor named "ShapeString_Pred", original data and standard input files for single and two-layer SVM model are accessible at an anonymous ftp site: ftp://cheminfo.tongji.edu.cn/BetaTurnPrediction/.

## Competing interests

The authors declare that they have no competing interests.

## Authors' contributions

ZT computed the features, generated the prediction model, performed experimental comparison and drafted the manuscript. TL participated in the design of the study and helped to draft the manuscript. RL helped with the preparation of the dataset and computed part of the features. WX and JS helped with the conception and design of the prediction method. YZ and GC participated in the design and coordination of the study. All authors have read and approved the final manuscript.

## References

[B1] Richardson JS The anatomy and taxonomy of protein structure Adv Protein Chem 1981 34 167 339 7020376 10.1016/s0065-3233(08)60520-3

[B2] Rose GD Gierasch LM Smith JA Turns in peptides and proteins Adv Protein Chem 1985 37 1 109 2865874 10.1016/s0065-3233(08)60063-7

[B3] Kabsch W Sander C Dictionary of protein secondary structure: pattern recognition of hydrogen-bonded and geometrical features Biopolymers 1983 22 12 2577 2637 10.1002/bip.360221211 6667333

[B4] Guruprasad K Rajkumar S Beta-and gamma-turns in proteins revisited: a new set of amino acid turn-type dependent positional preferences and potentials J Biosci 2000 25 2 143 156 10878855

[B5] Takano K Yamagata Y Yutani K Role of amino acid residues at turns in the conformational stability and folding of human lysozyme Biochemistry-Us 2000 39 29 8655 8665 10.1021/bi9928694 10913274

[B6] Chou PY Fasman GD Conformational parameters for amino acids in helical, beta-sheet, and random coil regions calculated from proteins Biochemistry-Us 1974 13 2 211 222 10.1021/bi00699a001 4358939

[B7] Wilmot CM Thornton JM Analysis and prediction of the different types of beta-turn in proteins J Mol Biol 1988 203 1 221 232 10.1016/0022-2836(88)90103-9 3184187

[B8] Wilmot CM Thornton JM Beta-turns and their distortions: a proposed new nomenclature Protein Eng 1990 3 6 479 493 10.1093/protein/3.6.479 2371257

[B9] Zhang CT Chou KC Prediction of beta-turns in proteins by 1-4 and 2-3 correlation model Biopolymers 1997 41 6 673 702 10.1002/(SICI)1097-0282(199705)41:6<673::AID-BIP7>3.0.CO;2-N

[B10] Chou KC Prediction of beta-turns J Pept Res 1997 49 2 120 144 9147309

[B11] Fuchs P Alix A High accuracy prediction of beta-turns and their types using propensities and multiple alignments Proteins-Structure Function and Bioinformatics 2005 59 4 828 839 10.1002/prot.20461 15822097

[B12] Altschul SF Madden TL Schaffer AA Zhang JH Zhang Z Miller W Lipman DJ Gapped BLAST and PSI-BLAST: a new generation of protein database search programs Nucleic Acids Res 1997 25 17 3389 3402 10.1093/nar/25.17.3389 9254694 PMC146917

[B13] Jones DT Protein secondary structure prediction based on position-specific scoring matrices J Mol Biol 1999 292 2 195 202 10.1006/jmbi.1999.3091 10493868

[B14] Pollastri G Przybylski D Rost B Baldi P Improving the prediction of protein secondary structure in three and eight classes using recurrent neural networks and profiles Proteins 2002 47 2 228 235 10.1002/prot.10082 11933069

[B15] Shepherd AJ Gorse D Thornton JM Prediction of the location and type of beta-turns in proteins using neural networks Protein Sci 1999 8 5 1045 1055 10.1110/ps.8.5.1045 10338015 PMC2144340

[B16] Kaur H Raghava G A neural network method for prediction of beta-turn types in proteins using evolutionary information Bioinformatics 2004 20 16 2751 2758 10.1093/bioinformatics/bth322 15145798

[B17] Kaur H Raghava G Prediction of beta-turns in proteins from multiple alignment using neural network Protein Sci 2003 12 3 627 634 10.1110/ps.0228903 12592033 PMC2312433

[B18] Kirschner A Frishman D Prediction of beta-turns and beta-turn types by a novel bidirectional Elman-type recurrent neural network with multiple output layers (MOLEBRNN) Gene 2008 422 1-2 22 29 10.1016/j.gene.2008.06.008 18598743

[B19] Kim S Protein beta-turn prediction using nearest-neighbor method Bioinformatics 2004 20 1 40 44 10.1093/bioinformatics/btg368 14693806

[B20] Zhang Q Yoon S Welsh WJ Improved method for predicting beta-turn using support vector machine Bioinformatics 2005 21 10 2370 2374 10.1093/bioinformatics/bti358 15797917

[B21] Pham TH Satou K Ho TB Prediction and analysis of beta-turns in proteins by support vector machine Genome Inform 2003 14 196 205 15706534

[B22] Liu LR Fang YP Li ML Wang CC Prediction of Beta-Turn in Protein Using E-SSpred and Support Vector Machine Protein Journal 2009 28 3-4 175 181 10.1007/s10930-009-9181-4 19488840

[B23] Cai YD Liu XJ Xu XB Chou KC Support vector machines for the classification and prediction of beta-turn types J Pept Sci 2002 8 7 297 301 10.1002/psc.401 12148778

[B24] Hu XZ Ll QZ Using support vector machine to predict beta- and gamma-turns in proteins J Comput Chem 2008 29 12 1867 1875 10.1002/jcc.20929 18432623

[B25] Guruprasad K Rajkumar S Beta-and gamma-turns in proteins revisited: a new set of amino acid turn-type dependent positional preferences and potentials J Biosci 2000 25 2 143 156 10878855

[B26] Kountouris P Hirst JD Predicting beta-turns and their types using predicted backbone dihedral angles and secondary structures BMC Bioinformatics 2010 11 (407) 10.1186/1471-2105-11-407 PMC2920885 20673368

[B27] Zheng C Kurgan L Prediction of beta-turns at over 80% accuracy based on an ensemble of predicted secondary structures and multiple alignments BMC Bioinformatics 2008 9 430 10.1186/1471-2105-9-430 18847492 PMC2613158

[B28] Liu LR Fang YP Li ML Wang CC Prediction of Beta-Turn in Protein Using E-SSpred and Support Vector Machine Protein Journal 2009 28 3-4 175 181 10.1007/s10930-009-9181-4 19488840

[B29] Kaur H Raghava G An evaluation of beta-turn prediction methods Bioinformatics 2002 18 11 1508 1514 10.1093/bioinformatics/18.11.1508 12424123

[B30] Shepherd AJ Gorse D Thornton JM Prediction of the location and type of beta-turns in proteins using neural networks Protein Sci 1999 8 5 1045 1055 10.1110/ps.8.5.1045 10338015 PMC2144340

[B31] Hutchinson EG Thornton JM PROMOTIF--a program to identify and analyze structural motifs in proteins Protein Sci 1996 5 2 212 220 8745398 10.1002/pro.5560050204 PMC2143354

[B32] Hobohm U Sander C Enlarged representative set of protein structures Protein Sci 1994 3 3 522 524 8019422 10.1002/pro.5560030317 PMC2142698

[B33] Fuchs P Alix A High accuracy prediction of beta-turns and their types using propensities and multiple alignments Proteins-Structure Function and Bioinformatics 2005 59 4 828 839 10.1002/prot.20461 15822097

[B34] Rost B Sander C Prediction of protein secondary structure at better than 70% accuracy J Mol Biol 1993 232 2 584 599 10.1006/jmbi.1993.1413 8345525

[B35] Cole C Barber JD Barton GJ The Jpred 3 secondary structure prediction server Nucleic Acids Res 2008 36 (Web Server issue):W197 W201 10.1093/nar/gkn238 PMC2447793 18463136

[B36] Montgomerie S Sundararaj S Gallin WJ Wishart DS Improving the accuracy of protein secondary structure prediction using structural alignment BMC Bioinformatics 2006 7 301. 16774686 10.1186/1471-2105-7-301 PMC1550433

[B37] Ison RE Hovmoller S Kretsinger RH Proteins and their shape strings. An exemplary computer representation of protein structure IEEE Eng Med Biol Mag 2005 24 3 41 49 15971840 10.1109/memb.2005.1436459

[B38] Kountouris P Hirst JD Prediction of backbone dihedral angles and protein secondary structure using support vector machines BMC Bioinformatics 2009 10 437 10.1186/1471-2105-10-437 PMC2811710 20025785

[B39] Wood MJ Hirst JD Protein secondary structure prediction with dihedral angles Proteins-Structure Function and Bioinformatics 2005 59 3 476 481 10.1002/prot.20435 15778963

[B40] Blayney JK Ojha PC Shapcott M Predicting three-dimensional structure of protein fragments from dihedral angle propensities and molecular dynamics Int J Comput Biol Drug Des 2010 3 2 146 163 10.1504/IJCBDD.2010.035240 20852338

[B41] Zhu Y Li T Li D Zhang Y Xiong W Sun J Tang Z Chen G Using predicted shape string to enhance the accuracy of γ-turn prediction Amino Acids in press 10.1007/s00726-011-0889-z 21424809

[B42] Li W Godzik A Cd-hit: a fast program for clustering and comparing large sets of protein or nucleotide sequences Bioinformatics 2006 22 13 1658 1659 10.1093/bioinformatics/btl158 16731699

[B43] Hovmöller S Zhou T Protein shape strings and DNA sequences http://www.fos.su.se/~pdbdna/pdb_shape_dna.html 10.2174/138920308785132703 18691122

[B44] Vapnik VN An overview of statistical learning theory IEEE Trans Neural Netw 1999 10 5 988 999 10.1109/72.788640 18252602

[B45] CC C CJ L LIBSVM:a library for support vector machines http://www.csie.ntu.edu.tw/~cjlin/libsvm

[B46] Matthews BW Comparison of the predicted and observed secondary structure of T4 phage lysozyme Biochim Biophys Acta 1975 405 2 442 451 1180967 10.1016/0005-2795(75)90109-9

[B47] Kakumani R Devabhaktuni V Ahmad M A two-stage neural network based technique for protein secondary structure prediction Conf Proc IEEE Eng Med Biol Soc 2008 2008 1355 1358 19162919 10.1109/IEMBS.2008.4649416

[B48] Nguyen MN Rajapakse JC Prediction of protein relative solvent accessibility with a two-stage SVM approach Proteins 2005 59 1 30 37 10.1002/prot.20404 15696542

[B49] Nguyen MN Rajapakse JC Prediction of Protein Secondary Structure with two-stage multi-class SVMs Int J Data Min Bioinform 2007 1 3 248 269 10.1504/IJDMB.2007.011612 18399074

[B50] Zhou T Shu N Hovmoller S A novel method for accurate one-dimensional protein structure prediction based on fragment matching Bioinformatics 2010 26 4 470 477 10.1093/bioinformatics/btp679 20007252

[B51] Kountouris P Hirst JD Prediction of backbone dihedral angles and protein secondary structure using support vector machines BMC Bioinformatics 2009 10 437 10.1186/1471-2105-10-437 20025785 PMC2811710

[B52] Amegbey G Stothard P Kuznetsova E Yee A Arrowsmith CH Wishart DS Solution Structure of MTH0776 from Methanobacterium thermoautotrophicum J Biomol Nmr 2005 33 1 51 56 10.1007/s10858-005-1275-5 16222557

[B53] Crooks GE Hon G Chandonia JM Brenner SE WebLogo: a sequence logo generator Genome Res 2004 14 6 1188 1190 10.1101/gr.849004 15173120 PMC419797

